# Host-induced silencing of the *CpCHI* gene resulted in developmental abnormalities and mortality in maize stem borer (*Chilo partellus*)

**DOI:** 10.1371/journal.pone.0280963

**Published:** 2023-02-06

**Authors:** Olawale Samuel Adeyinka, Idrees Ahmad Nasir, Bushra Tabassum

**Affiliations:** 1 Centre of Excellence in Molecular Biology, University of the Punjab, Lahore, Pakistan; 2 Department of Chemistry, Physics and Atmospheric Sciences Jackson state University, Jackson, MS, United States of America; 3 School of Biological Sciences, University of the Punjab, Lahore, Pakistan; Gomal University, PAKISTAN

## Abstract

RNAi-based insecticides for crop protection have witnessed rapid improvement over the years. However, their potential to efficiently control maize stem borer (*Chilo partellus*) pests has remained underexplored. In this study, double-stranded *C*. *partellus* chitinase (*dsCHI*) toxicity was investigated in *C*. *partellus* larvae. Furthermore, we developed transgenic maize lines expressing dsRNA targeted against *C*. *partellus* chitinase transcripts and performed detached leaf insect feeding bioassays. Our results revealed that *C*. *partellus* chitinase transcript expression was significantly downregulated by 57% and 82% in the larvae. Larvae exhibited various phenotypic distortion levels across developmental stages, and 53% mortality occurred in transgenic fed larvae compared to those fed on nontransgenic leaves. In conclusion, we have identified the *C*. *partellus* chitinase gene as a potential target for RNAi-mediated control and demonstrated that oral delivery via bacteria and plant-mediated delivery are viable means of achieving *C*. *partellus* RNAi-mediated control.

## Introduction

Maize is an important staple food worldwide and is widely used in livestock feed formulation. It is an essential crop with the potential to contribute significantly to achieving the second goal (Zero Hunger) of sustainable development. Its importance extends to its utilisation as a vital renewable energy source. The insurgence of maize stem borers (*C*. *partellus*) is a serious threat that hampers maize optimal yield productivity. *C*. *partellus* larvae feed inside maize stems, which results in ineffectiveness in the chemical control of *C*. *partellus*. Consequently, most local farmers abuse chemical usage. Over time, such abuse of pesticides results in pest resurgence, an outbreak of secondary pests, serious effects on biodiversity and beneficial organisms, and high risks to the environment and human health [1]. RNA interference (RNAi) technology is a well-known ecological and economic strategy to control insect pests to obtain a sustainable food supply. Furthermore, the application of exogenous RNA delivery approaches has been proven to be a viable means of managing agricultural pests [2–4].

RNAi technology is commonly employed to study gene functions and is currently used as an alternate control measure for agricultural pests. However, several factors, such as inadequate delivery methods, poor RNAi efficiency, and dsRNA degradation, greatly influence RNAi application in insect pest control. The challenges of identifying the specific gene suitable for the control of targeted insect pests limit its extensive usage in agricultural pest control. Undoubtedly, there are successful reports in several insect orders with limited success in Lepidoptera. This study focuses on viable host-induced gene silencing (HIGS) while targeting chitinase as a potential target. Chitinases are members of the *O*-glycoside hydrolase superfamily [5], which hydrolyse chitin at intermediate regions to produce oligomers of NAcGlc. Chitinases have been reported to function in moulting fluid and gut tissues [6–8]. Additionally, chitinases facilitate the digestion of chitin in the exoskeleton and peritrophic membrane (PM) [9, 10]. Genes encoding insect chitinases have been identified and characterised from some lepidopterans [11–15] but are limited to *C*. *partellus*.

Previous studies have shown that suppressing chitinases and chitin synthase affects the development of various insects and suggest that it is important to control insects [16–20]. Transgenic tobacco plants that express double-stranded chitin synthase further support its potential as a significant target for *C*. *partellus* control [21]. In this study, we described a novel chitinase gene in *C*. *partellus* and its expression pattern at all developmental stages. Furthermore, we evaluated the knockdown effect of bacterially expressed double-stranded *C*. *partellus* chitinase (*dsCHI*) and purified *dsCHI* of *C*. *partellus* genes. Finally, we demonstrated *C*. *partellus* RNAi-mediated control through maize transgenic plants expressing *dsCHI* targeting *C*. *partellus* chitinase.

## Materials and methods

### Insect management

Maize stem borer samples were taken from the maize experimental plot and transferred to the insectary facility (26 ± 2°C under a 14:10 h light:dark cycle and 65±5% relative humidity) at the Centre of Excellence in Molecular Biology, Pakistan. The larvae were maintained on an artificial diet [22].

### Cloning of chitinase for dsRNA synthesis

Total RNA was extracted from larvae at different developmental stages using TRI Reagent (Sigma‒Aldrich, St. Louis, USA) according to the manufacturer’s instructions. The DNA impurities were removed from the total RNA with DNaseI (#EN0521; Thermo Scientific), and 1 μg of the RNA was reverse transcribed by a RevertAid First Strand cDNA synthesis kit (#K1622; Thermo Scientific) according to the manual instructions. The primer pairs (*CHI*) used are presented in Table 1 and were designed to amplify the chitinase gene (Accession number; MK560453.1) without off-target potential using dsCheck online software [23]. The reaction mixture (20 μl) for amplification contained 2 μl of 10X Taq Buffer, 0.4 mM MgCl_2_, 0.15 mM dNTP, 1.25 U of Taq, 0.5 μM of both forward and reverse primers, and 1 μl of cDNA. The amplification was performed according to the following cycling profile: initial denaturation at 95°C for 5 min, followed by 35 cycles of 95°C denaturation for 30 s, 58°C annealing for 30 s, and 72°C extension for 30 s. The amplified products were ligated into the pCR2.1 vector and transformed into *E*. *coli* top 10 competent cells. Positive clones were confirmed through restriction digestion (*EcoR1)* and then sequenced.

**Table 1:** List of primers used in the study.

Gene name	Primers (5’-3’)
Beta-tubulin	F: GTCGTAGAACCGTACAAC R: CGGAAGCAGATGTCATAT
Eukaryotic translation elongation	F: AGGAAATCAAGAAGGAAGTATCC R: CAAGGCATTTTGGTTGAAGG
*CHI*	F: AAGCTTCTCCGGTGTTGGTGTAGTATG R: TCTAGAAACGACGGTCTCAAGTTATGG
*dsCHI*	F: TCGTAATAAGCCCAGAATC R: GTAACAATAACTACGGACTC
*dsEGFP*	F: AAGCTTAAGGGCGAGGAGCTGTTCACCG
	R: TCTAGACAGCAGGACCATGTGATCGCGC

The underline nucleotides are the restriction enzymes (Hind III and Xbal) added to the primers to facilitate cloning.

### Phylogenetic analysis of chitinase sequence

The sequenced nucleotide was compared with other chitinase nucleotides in the National Center for Biotechnology Information (NCBI) by BLASTn (http://www.ncbi.nlm.gov/BLAST). The molecular weights and theoretical pIs were predicted using the Compute pI/Mw tool (https://web.expasy.org/compute_pi/). The Clustal Omega multiple sequence alignment program online (https://www.ebi.ac.uk/Tools/msa/clustalo) was used for the alignment. The amino acid sequences were predicted by EMBOSS Transeq (http://www.ebi.ac.uk/Tools/st/emboss_transeq). The predicted amino acid relatedness with other Lepidoptera was used for the phylogenetic analysis using MEGA X [24] with 1000 bootstrap values.

### dsRNA synthesis and purification

The amplified chitinase product as well as EGFP (enhanced green fluorescent protein) amplified from pcDNA3-EGFP were ligated at the Hind III and Xbal restriction sites of the L4440 vector and transformed into the *E*. *coli* HT115 strain. Positive *E*. *coli* HT115 colonies were grown overnight in 5 ml YT media (yeast extract (10 g/L), Bacto peptone (5 g/L), NaCl (10 g/L)) containing 100 μg ml−1 ampicillin at 37°C. dsRNA synthesis was induced with 0.6 mmol l−1 IPTG (isopropyl β-D-1-thiogalactopyranoside) at 37°C for another 3–4 hours, and the dsRNA was purified as described by [25, 26]. Briefly, induced bacterial cells were pelleted and resuspended in 10 mM EDTA, 1 M ammonium acetate; then, an equal volume of phenol:chloroform:isoamyl alcohol (25:24:1) was added, and the suspension was vortexed. The samples were incubated at 65°C for 30 min and centrifuged at 10,000 × g for 20 min. The cleared upper phase was transferred to new 50 ml tubes containing equal amounts of isopropanol and incubated at −20°C overnight. The nucleic acid precipitates were pelleted at 10,000 *rpm* for 20 min and immediately treated with 0.4 U/μl DNase and 0.2 μg/μl RNase A for 30 min.

### Insect bioassay

To estimate the relative transcription of chitinase in *C*. *partellus*, samples were collected across developmental stages from three technical replicates and two independent biological replicates. First-instar larvae were used as reference samples for the temporal expression profiling analyses. Beta-tubulin was used as an internal control for the normalisation of transcript abundance across developmental stages based on initial findings [25].

To evaluate the survival of 3^rd^ instar larvae, 200 μl of bacterially induced dsRNA as reported by [27] was compared with 15 μg of purified dsCHI. Previous studies have shown that lepidopterans are susceptible to high concentrations of dsRNA [28]. L3 larvae were fed diets overlaid with the treatments for 12 days. Three biological replicates, each comprising 30 larvae/rep, were used for the feeding assay, and nuclease-free water and empty HT115 were used as a control treatment. The relative survival rates among the dsRNA-fed and control-fed larvae were analysed by Kaplan‒Meier survival analysis using GraphPad Prism software.

To monitor chitinase suppression, 200 μl of dsCHI-expressing bacteria, empty vector-expressing HT115 *E*. *coli* and dsEGFP-expressing bacteria were overlaid on rectangular artificial diet pellets [22] and fed to starved 3rd instar larvae [29] for 15 consecutive days. The experiment comprised three technical replicates and three biological replicates (30 larvae per treatment). Total RNA from three larvae per replicate was extracted by TRIzol and reverse translated for RT−qPCR to evaluate the mRNA abundance of chitinase at different time intervals. ELF was used as an internal reference for the normalisation of transcript abundance [25]. Data were taken on larval body weight as well as the observation of morphological changes in each developmental stage.

### Construction of the *dsCHI* hairpin and its expression in maize

**Fig 1:**
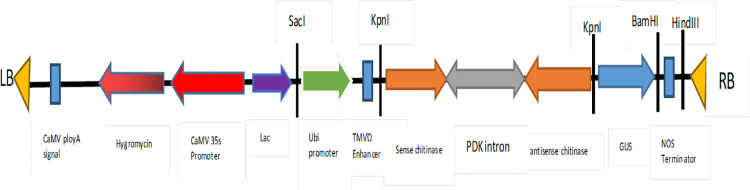
Schematic illustration of the T-DNA vector used for maize transformation. *Pyruvate orthophosphate* dikinase (PDK) intron of 767bp introduced between the sense and antisense chitinase sequence was driven by poly Ubi maize promoter and TMV omega enhancer to increase transgene expression.

The plant transformation binary vector pCAMBIA1300, modified to include the *maize ubiquitin* (*Ubi*-1) promoter and renamed pSVP [30], was used to drive dsCHI expression. To construct dsCHI hairpin cassettes (Fig 1), a pyruvate orthophosphate dikinase (PDK) intron of 767 bp was introduced between the sense and antisense coding sequence of 501 bp of the *Chilo partellus* chitinase gene (MK560453) and synthesized by Bio Basic Inc. The cassette was introduced at the Kpnl restriction site of pSVP and transformed into competent Agro*bacterium tumefaciens* LBA4404 made by a Bio-Rad electroporator. One hundred microlitres of transformed cells was spread on YEP agar containing 25 μg/ml tetracycline and 50 μg/ml kanamycin and grown at 28°C for 48 hours. The positive clones were confirmed by colony PCR with dsCHI primers (Table 1) designed to amplify a 279 bp region of the *dsCHI* hairpin and streaked on YEP agar for subsequent infection. Transformation was performed by the immature maize embryo transformation protocol described by [31]. The transformants were maintained on media containing 100 mg/ml cefotaxime and 200 mg/ml carbenicillin to grow until 4 foliage leaves emerged. Then, they were moved into sterile pot soil and transferred to a screen house (S1 Fig in S1 File). Genomic DNA was isolated from the putative transformants and untransformed control for PCR confirmation.

### Determination of ß-glucuronidase (GUS) activity in positive transformants

The GUS histological assay described by Jefferson *et al*., [32] was performed on the germinating embryo as well as the spikelet of the transgenic and nontransgenic maize. The solution mix consisted of 1 mg/mL X-Gluc (5-bromo-4-chloro-3-indolyl-β-D-glucuronide) substrate, 100 mM sodium phosphate buffer (pH 7.0), 5 mM K3Fe (CN)6, 5 mM K4Fe (CN)6, 0.5 mM EDTA (ethylenediaminetetraacetic acid), 0.1% Triton X-100, and 20% methanol. The germinating embryo and the spikelet were incubated in the solution mix overnight at 37°C. Then, the samples were bleached in 80% ethanol for approximately 48 hours to observe blueish colouration.

### Dot blot hybridisation and southern blot analysis

Seven transgenic T0 plants were used for dot blot hybridisation and southern blot analysis. For dot blot hybridisation, 15 μg genomic DNA of both transgenic and nontransgenic maize was denatured at 99°C for 10 minutes and immediately cooled on ice for 5 minutes. Ten microlitres of the denatured samples was gradually spotted on a nylon membrane (Amersham Hybond-Nx) and allowed to dry. Similarly, genomic DNA (20 μg) of both transgenic and nontransgenic maize plants was digested with the KpnI restriction enzyme and used for southern blot analysis. The digested samples were hybridised with a chitinase probe (501 bp) that was prepared by a Biotin DecaLabel DNA labelling kit (#KO651 ThermoFisher Scientific) according to the manufacturer’s protocol. The Biotin chromogenic detection kit (cat#K0661) was used to detect biotin-labelled dsCHI according to the manufacturer’s protocol.

### Transgenic expression of *dsCHI hairpin* and evaluation of chitinase transcript suppression in *C*. *partellus* larvae

To examine the expression of *dsCHI* in T1 transgenic plants, the total RNA used for cDNA synthesis was isolated with a PureLink® RNA Mini Kit according to the manufacturer’s protocol and reverse transcribed. To investigate the knockdown of *C*. *partellus* chitinase after feeding on transgenic maize leaves and nontransgenic maize leaves, RNA was isolated from two biological replicates comprising three larvae. Elongation factor protein (ElF) was used as an internal reference for the normalisation of transcript abundance [25]. The reaction mixture (10 μl) contained Maxima SYBR Green qPCR 2X Master mix (Thermo Scientific), 500 nM each of the forward and reverse primers (ds*CHI)*, 0.5 μl cDNA, and nuclease-free water. Amplifications were performed with the following cycling profile: initial denaturation at 95°C for 5 min followed by 35 cycles of denaturation at 95°C for 30 s, annealing at 58°C for 30 s, and extension at 72°C for 30 s. Gene expression analysis was obtained through the Livak method [33].

Furthermore, a bioassay was conducted on the T1 transgenic maize line with the highest expression of dsCHI compared to that of the nontransgenic maize. A total of 40 second instar larvae were fed transgenic leaves (four replicates of 10 larvae each). Control larvae were fed with nontransgenic maize leaves. Data were recorded for weight gained by the insect, surface consumption of the leaves by insect and number of dead larvae.

### Statistical analysis

The transcript data and bioassay data from experimental replicates were analysed using GraphPad Prism software for one-way analysis of variance (ANOVA) with Tukey’s post hoc t test to determine differences. A p value < 0.05 was considered statistically significant.

## Results

### Phylogenetic analysis of chitinase

**Fig 2:**
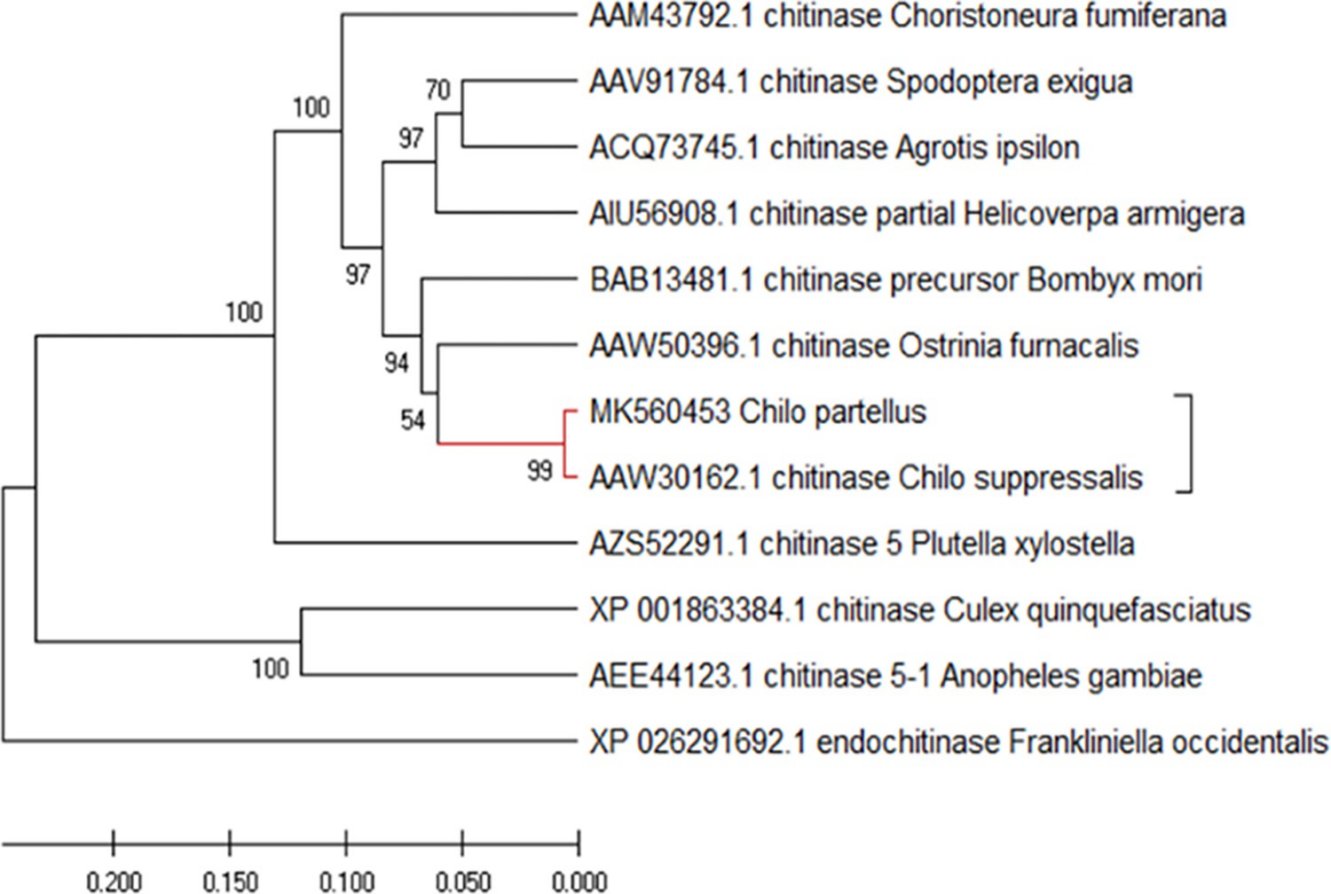
Phylogenetic tree analysis showing the relationship of *C*. *partellus* chitinase genes among lepidopteran chitinase. Analysis was based on the neighbour-joining method according to amino acid sequences using MEGA X. Bootstrap support values with 1,000 samples are shown on the branches.

The cDNA sequence contains 501 nucleotides, encoding 169 amino acid residues with a molecular mass of 18.75 kDa and a theoretical isoelectric point (pI) of 5.82. The C. partellus chitinase multiple sequence alignment with other lepidopteran orders from NCBI indicated that most of the nucleotides were highly conserved. *C*. *partellus* chitinase had the highest similarity of 88% with *C*. *suppressalis* chitinase. The phylogenetic tree (Fig 2) generated three major clusters, and most of the chitinase genes from the different lepidopterans fell in the same cluster, depicting relatedness among the lepidopterans that were used in this study. *C*. *suppressallis* is the closest lineage to C. partellus among the Lepidoptera with a strong bootstrap.

### Temporal expression pattern of chitinase in *Chilo Partellus*

Chitinase mRNA was detected in all developmental stages from first instar larva to adult. The lowest expression was found in the L1 stage, whereas L3, L5 and pupa had moderate expression, and the highest expression was in L2, L4 and adults (S2 Fig in S1 File). The functional properties of chitinase are optimal during specific (L2, L4 and adult) developmental stages, which suggests that L2 is the best stage to initiate silencing of chitinase mRNA in *C*. *partellus*.

### dsCHI synthesis via IPTG induction

We assembled a dsRNA-expressing vector by ligating sense and antisense coding sequences of 501 bp at the Hind III and Xbal restriction sites of the L4440 vector. The ligation was confirmed through restriction digestion; one of ~2.7 kb transcripts corresponded to the L4440 vector, while the second ~500 bp transcript corresponded to the transgene (S3A Fig in S1 File). The recombinant vectors were transformed into *E*. *coli* strain HT115, which lacked double-strand-specific RNaseIII activity. T7 RNA polymerase activity was induced using 0.6 mM IPTG. Total bacterial RNA was extracted, and the presence of long dsRNA segments in total RNA was analysed (S3B Fig in S1 File).

**Fig 3:**
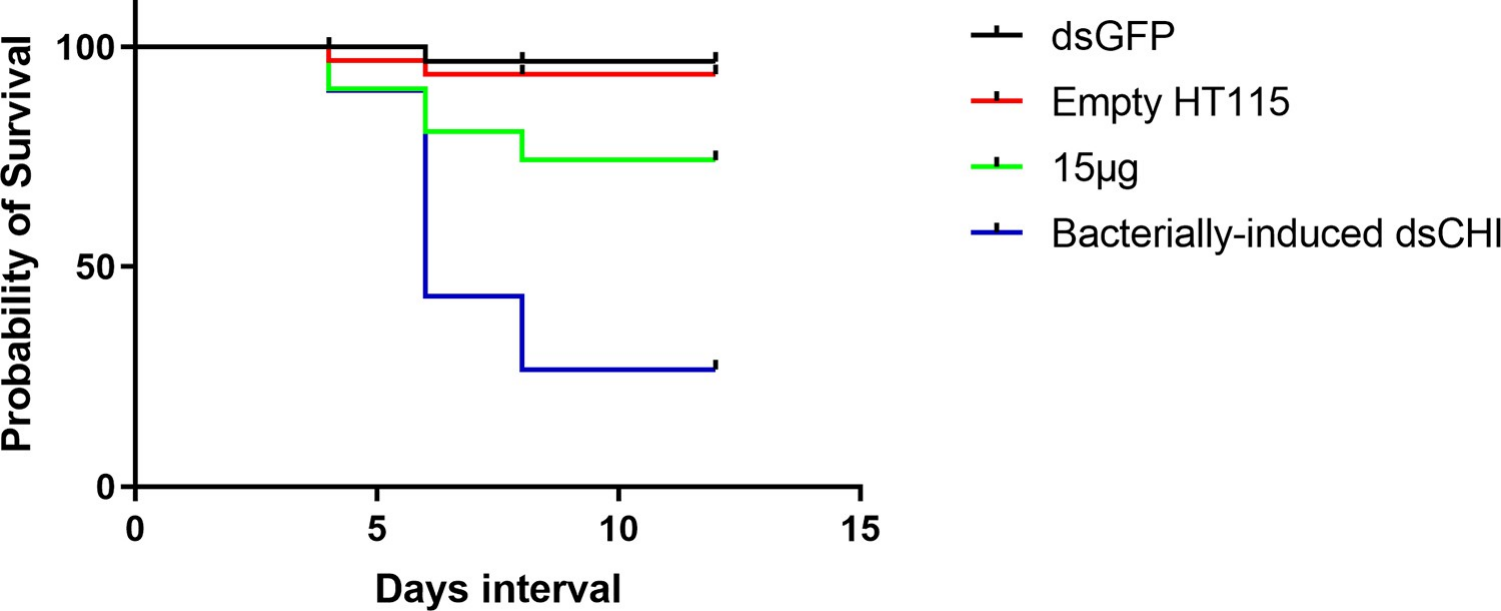
Kaplan-Meier analysis indicating survival after feeding purified *dsCHI* and bacterially-induced *dsCHI* to *C*. *partellus* larvae.

Survival assays on 3^rd^ instar larvae indicated variable susceptibility between the purified dsRNA and bacterially expressed dsRNA. The survival rate of the control larvae after 12 days was relatively close to 100%. There was a significant change in the survival rate of purified dsRNA and bacterially expressed dsRNA compared to the two controls (dsGFP and empty vector HT115). Third instar larvae treated with 15 μg of purified dsRNA exhibited moderate percent survival, whereas bacterially expressed dsRNA displayed the lowest percent survival (Fig 3) compared to the control after 12 days of exposure (Log-rank Mantel‒Cox, *p*< 0.001).

### *dsCHI*-expressing bacteria induced chitinase suppression and morphological deformity

**Fig 4:**
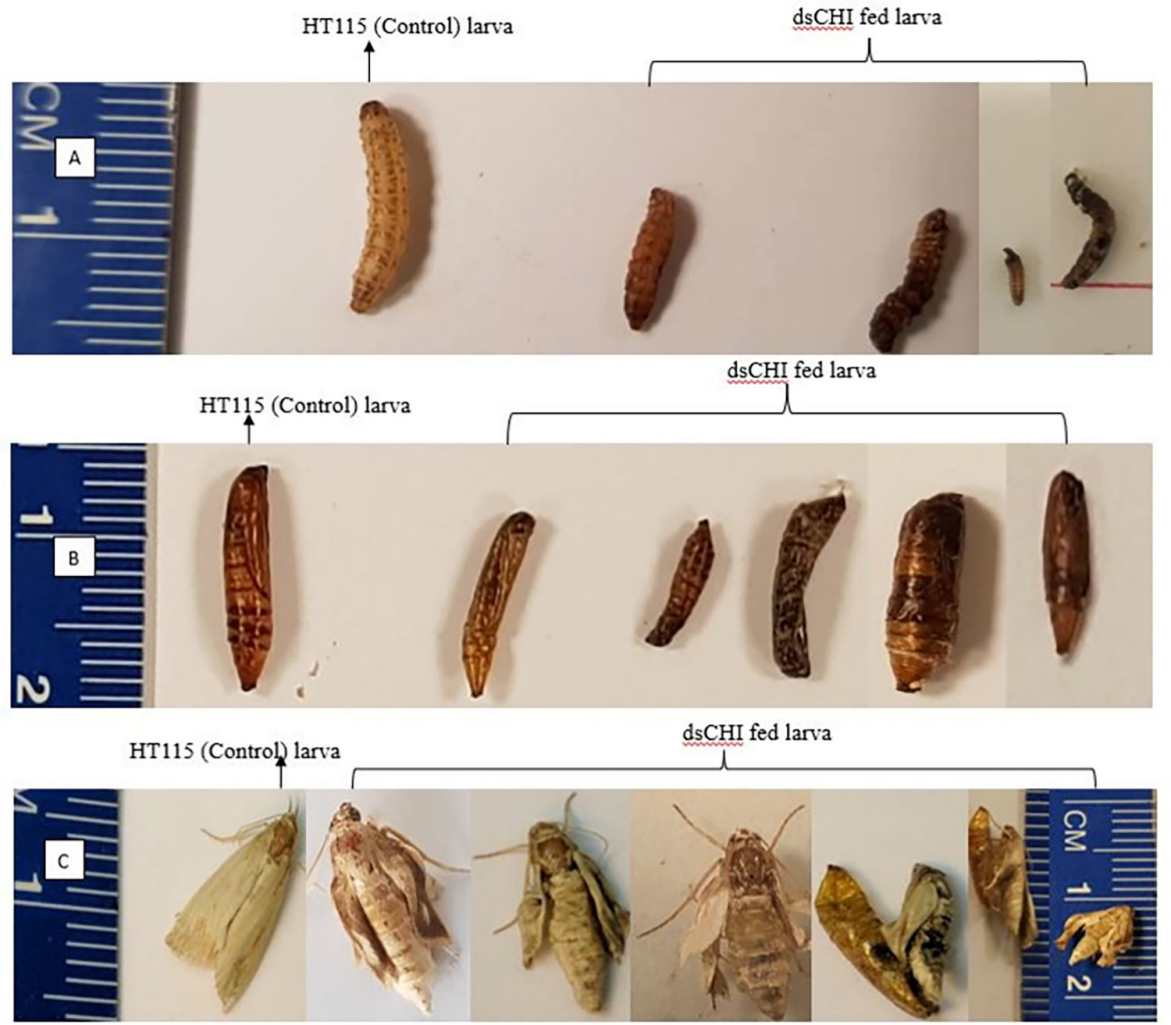
The effect of *dsCHI* on the various phenotype (A) various deformed shapes of dsRNA fed larvae compared to those feed without dsRNA (control), (B) different morphological deformity observed in some pupa from dsRNA fed larvae compared to the normal pupa (control), C) phenotype abnormality resulted from dsRNA fed larva compared to larvae that was fed with control.

Several deformed larval phenotypes were observed during their exposure to chitinase dsRNA (Fig 4A). Most of the deformed larva eventually died. However, some larva managed to metamorphize into a pupa (Fig 4B) but failed to emerge as an adult, and when this occurred, several deformed phenotypes were observed across the treatments (Fig 4C). Oral exposure to bacterially expressed *dsCHI* effectively induced phenotypic distortion in larvae, pupae, and emerging adults of *C*. *partellus*.

**Fig 5:**
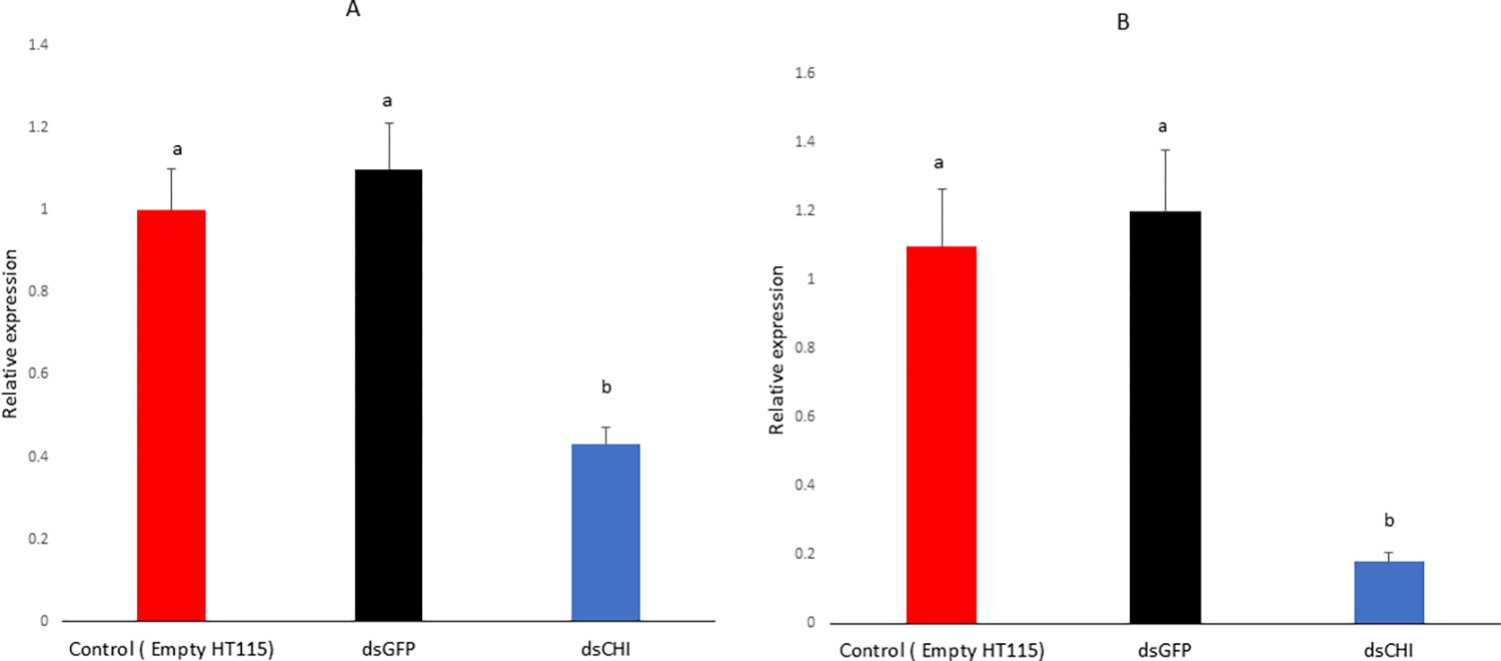
Relative knockdown in transcript levels of chitinase gene during in-vitro feeding assay with bacterially expressed *dsCHI* after (A) period of 5 days post-exposure. (B) period of 15 days post-exposure. Values are expression mean ±standard error and different letters indicate significant different (p<0.05) between *dsCHI* gene and the controls (Empty *HT115* and *dsGFP*).

RT‒qPCR analysis revealed that the mRNA abundance of chitinase decreased by 57% and 82% at 5 and 15 days postexposure to bacterially expressed dsRNA compared to the controls (Fig 5). Overall, the knockdown percentages for chitinase genes were more pronounced and significant at 15 days of exposure to bacterially expressed dsRNA than at 5 days of exposure. This indicated that the longer exposure time of bacterially expressed dsRNA plays an essential role in the RNAi response in *C*. *partellus*. A significant reduction in larval net body weight was observed after 15 days of dsCHI exposure.

### Cloning and confirmation of dsCHI in pCAMBIA-UBI 1300

The synthesized *dsCHI* gene was isolated from the pUC57 vector (S4A Fig in S1 File) and ligated into the pCAMBIA1300 binary vector at the KpnI restriction site. The confirmed clone of *dsCHI* in pCAMBIA1300 was named Pcambia1300-dsCHI, and it revealed two distinct restricted fragments; one of ~10 kb depicting pCAMBIA1300 and one of ~1650 bp depicting the dsCHI gene (S4B Fig in S1 File). The Pcambia1300-dsCHI construct was transfected into Agrobacterium cells, and the transgene *dsCHI* insertion was verified by amplification with gene chitinase-specific primers. Amplification of a ~279 bp fragment indicates positive Agrobacterium clones harbouring the transgene (S4C Fig in S1 File).

### PCR confirmation of transgene maize expressing *C*. *partellus* chitinase

**Fig 6:**
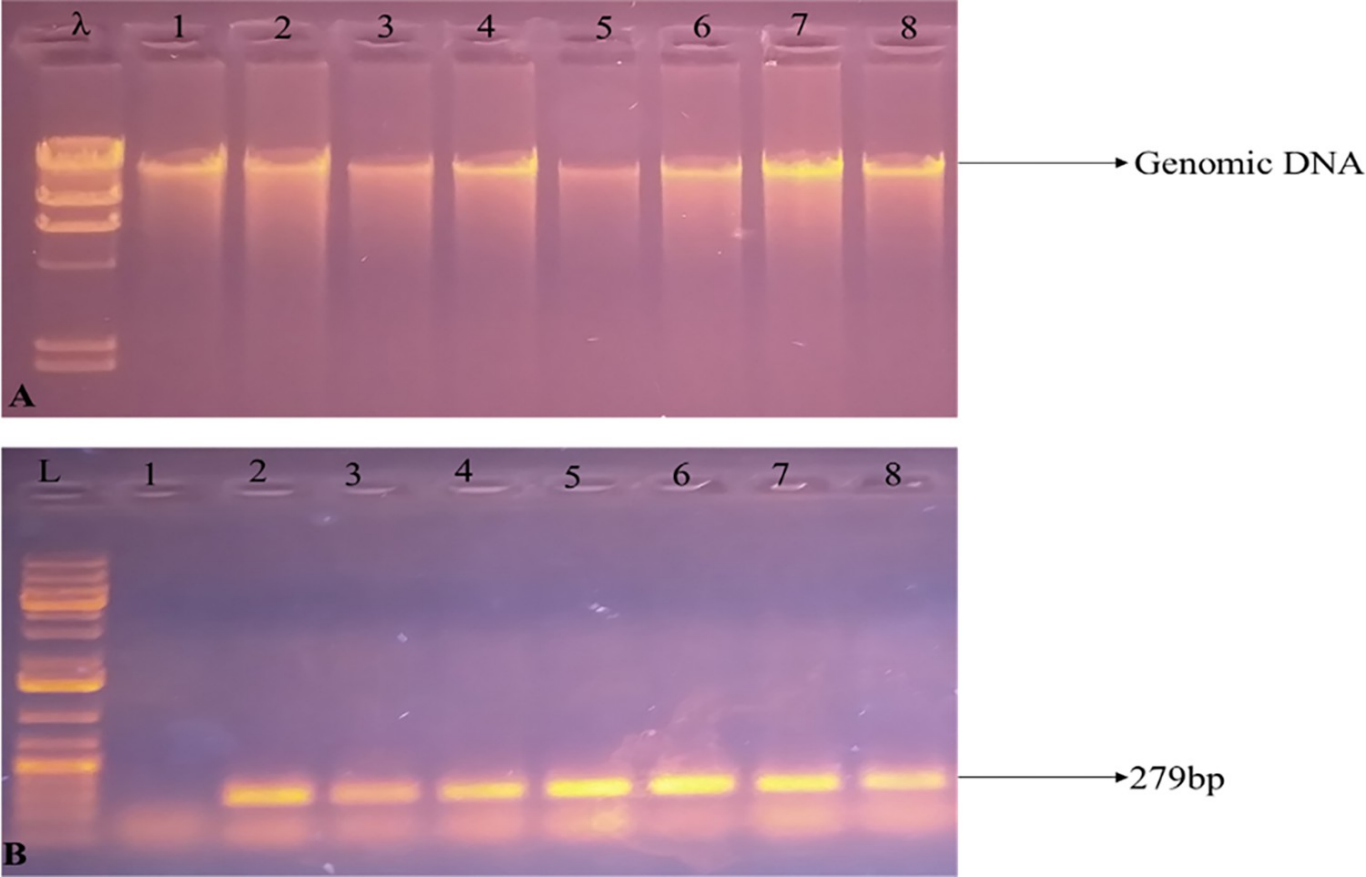
Maize genomic DNA and amplification of *dsCHI*. (A) λ indicate lambda ladder and 1–8 lanes indicated quality of genomic DNA isolated from Maize. (B) L is 1kb plus ladder and 1 lane does not show amplification from non-transgenic Maize while 2–8 lanes showed the PCR amplification of genomic DNA from 7 events.

Very high-quality genomic DNA was isolated from maize leaves, as shown in Fig 6A, and transgene insertion was verified through PCR amplification with gene-specific primers. Out of a total of 500 regenerated maize plants, only seven plants were positive (Fig 6B). Based on this, the overall transformation efficiency was 1.4%. The potential positive maize plants of the T_0_ generation were tagged properly for further analysis.

**Fig 7:**
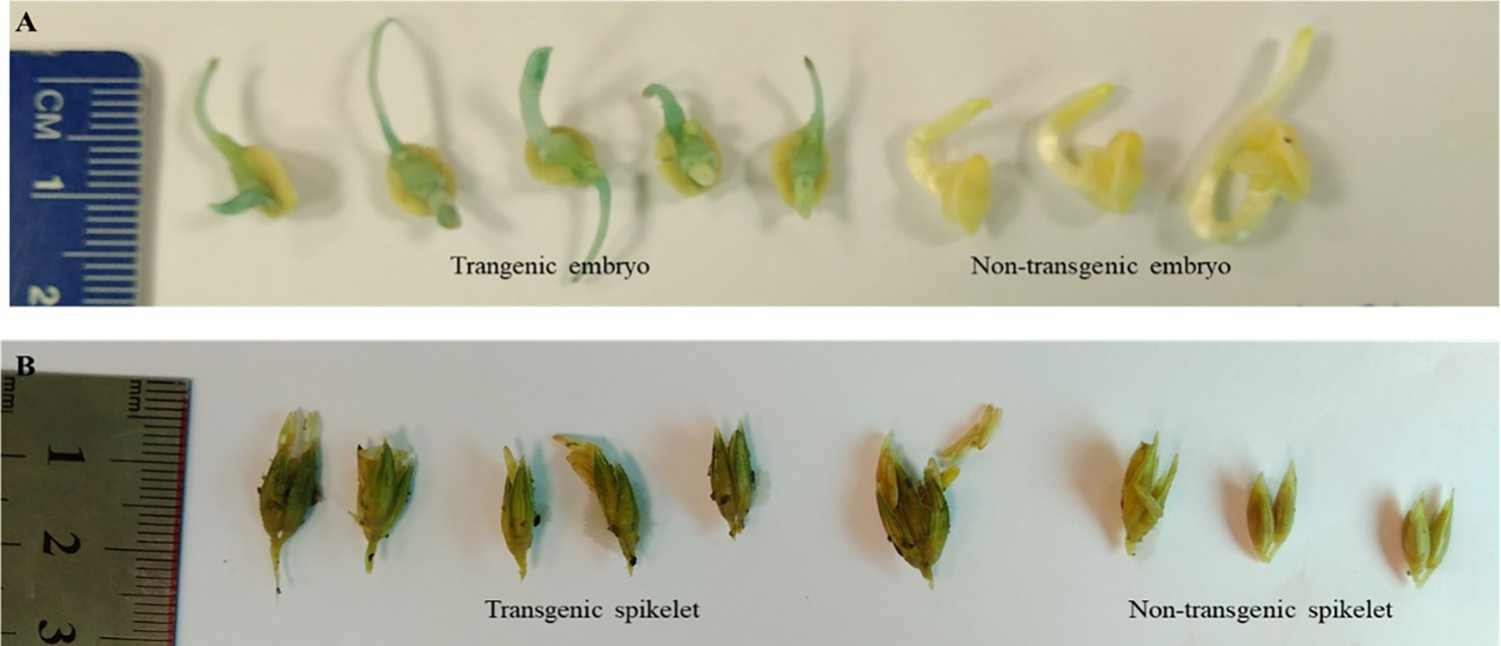
Histochemical staining of transgenic and control maize. (A) Blue colour indicating the presence of GUS gene in germinated embryo. (B) Blue-like colour indicated the presence of GUS gene in spikelets.

A histochemical *GUS* screening assay was performed on the germinating embryos. The germinating transformed embryos developed a bluish colour when stained with *GUS* substrate, while no colour was visible in nontransgenic maize embryos that were treated in parallel (Fig 7A). Similar findings were observed when the spikelets from the initial confirmed transformed maize plantlets were stained, as shown in Fig 7B. The blue colour-specific *GUS* expression was visible in transgenic maize samples, while no expression in the form of colour was observed in nontransgenic maize samples.

### Transgene integration studies by dot blot and southern blot analyses

The genomic integration of the transgene *dsCHI* was revealed through dot blot and Southern blot assays. All seven PCR-positive maize plants showed chromogenic reactions upon hybridisation with a biotin-labelled probe, while no signal was detected in the control, nontransgenic sample (S5A Fig in S1 File). Similar findings were found when digested genomic DNA of transformed maize plants was hybridised with a biotin-labelled probe, and a strong signal was detected in all transgenic maize samples depicting positive integration of T-DNA in the maize genome. The nontransgenic maize sample did not exhibit any hybridisation signal (S5B Fig in S1 File).

**Fig 8:**
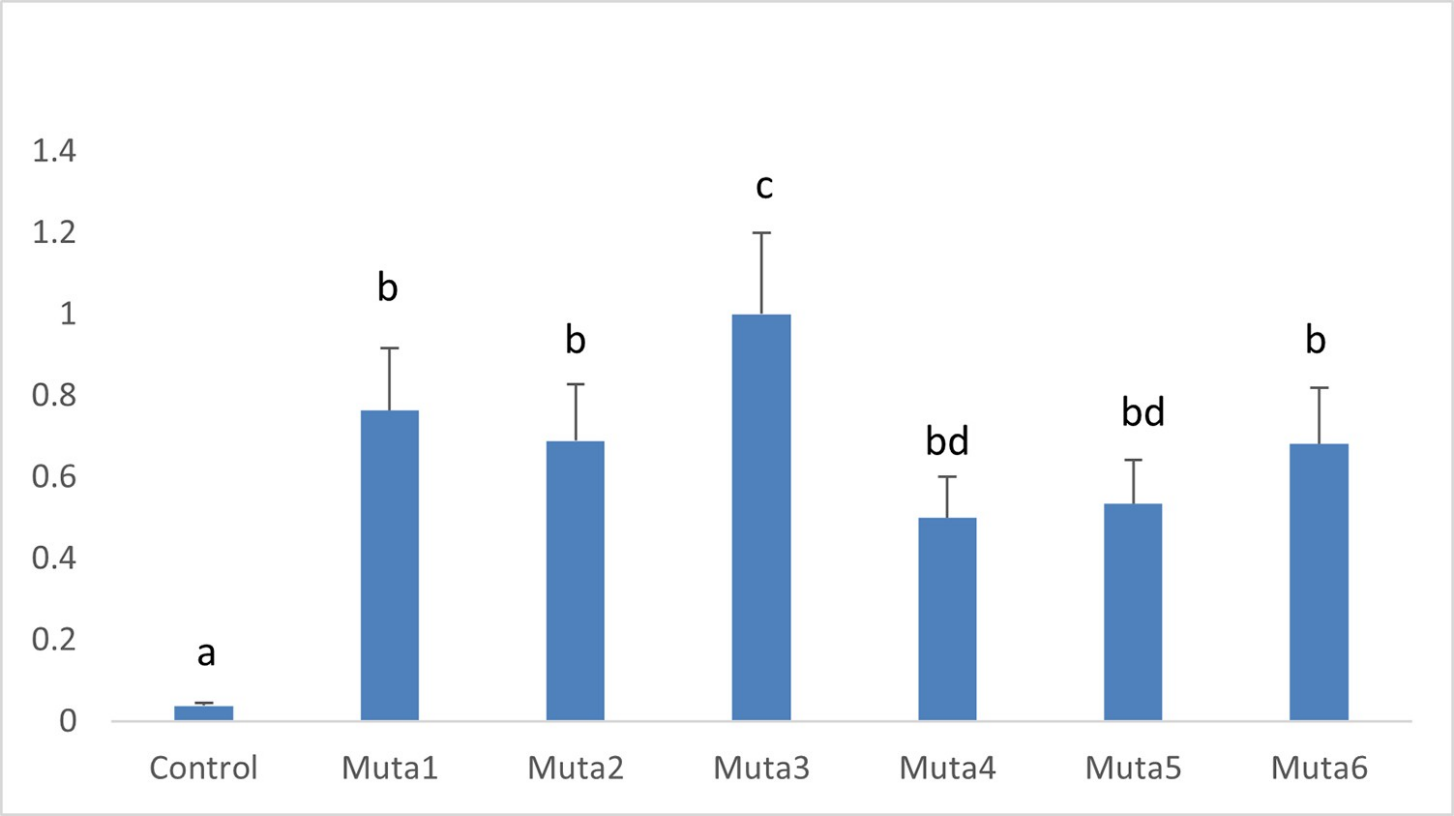
RT-qPCR analysis of T1 transgenic maize plants. Muta1—Muta6 indicating different transgenic lines. Values are expression mean ± standard error and different letter indicate significant different (p < 0.05).

To evaluate the *dsCHI* transcript levels in transgenic maize lines, quantitative real-time PCR (qRT‒PCR) analysis was performed. Variable mRNA expression of dsCHI was revealed in transgenic lines (Muta1, Muta2, Muta3, Muta4, Muta5 and Muta6). However, the nontransgenic control maize line exhibited nonsignificant expression. The highest mRNA expression of *dsCHI* was obtained in the transgenic maize line Muta3 (Fig 8).

### Bioassay analysis of *C*. *partellus* larvae feeding on T_1_ transgene maize

**Fig 9:**
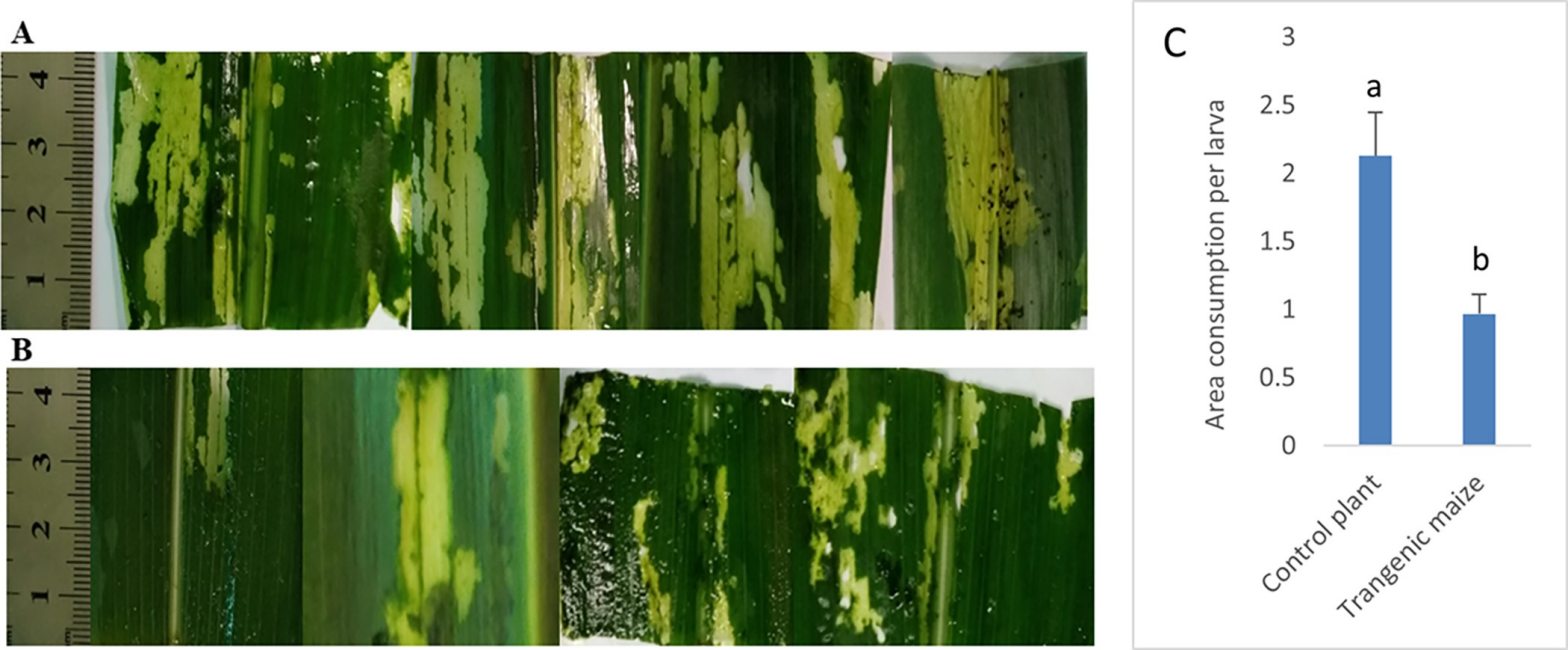
(A) Indicating the area non-transgenic maize consume larva (B) indicated the area of transgenic maize consume by larva (C) illustrating the quantification of the area consumed per larva at 4 days of feeding.

The transgenic maize line Muta4, having the highest transgene expression, was evaluated for insect resistance through an in vitro feeding assay. The area of consumption by *C*. *partellus* larvae in both transgenic and nontransgenic maize leaves after 5 days of infestation was quantified using ImageJ software. There was a significant difference in consumption area among nontransgenic (Fig 9A) leaf samples compared to transgenic leaves (Fig 9B). Briefly, the area consumed in the control nontransgenic leaves was 21.^29 cm2^ (approximately 2.1 cm^2^ per larva), while the area consumed in the transgenic leaves was 9.68 cm^2^ (approximately 0.97 cm^2^ per larva) (Fig 9C).

### *dsCHI* mRNA expression in the transgenic maize line correlates with increased mortality in *C*. *partellus* larvae

**Fig 10:**
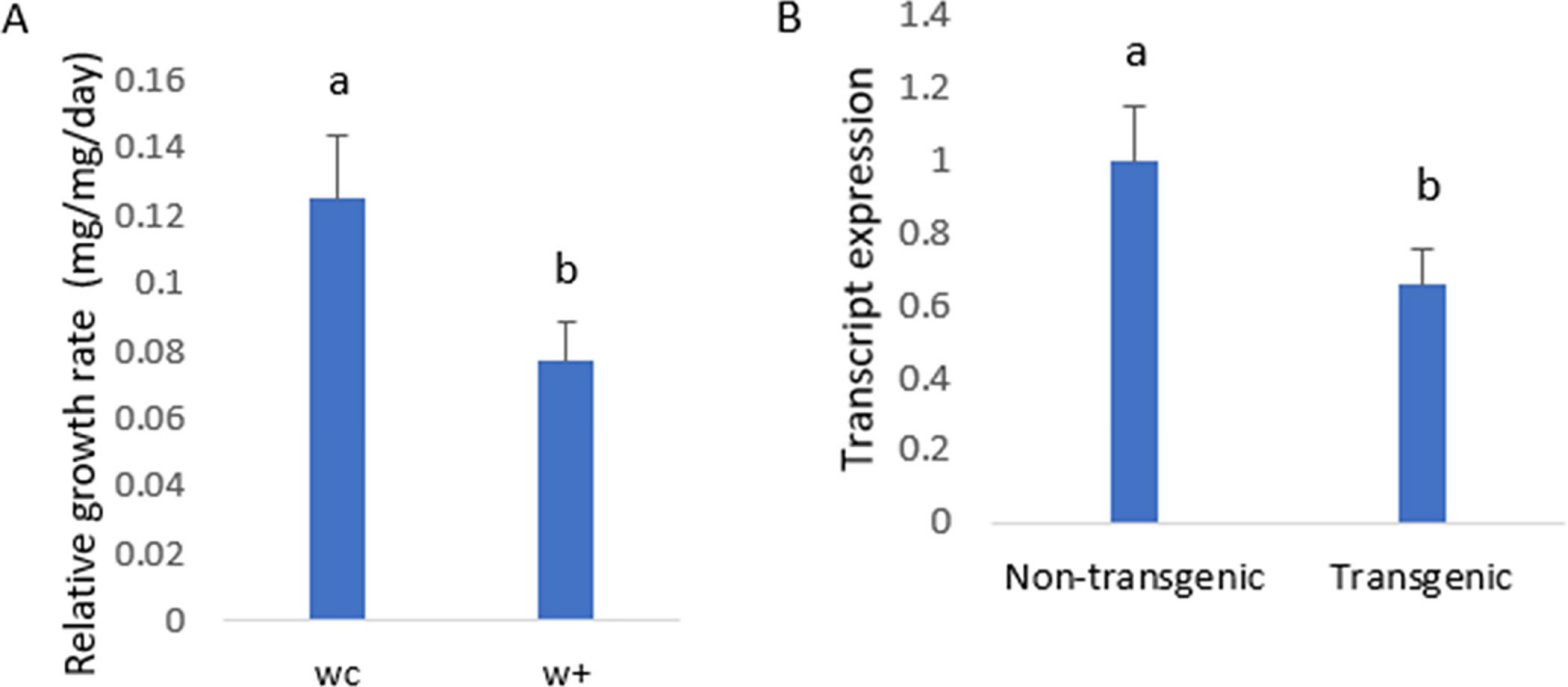
(A) Relative growth rate comparison estimates between larvae that were fed with non-transgenic Maize (wc) and transgenic maize leaves(w+) (B) *dsCHI* transcript abundance in *C*. *partellus* larvae after *dsCHI*-transgenic and non-transgenic post feeding. values are mean ±standard error and different letters indicate significant different (p<0.05).

To investigate the effect of dsCHI on the larval growth rate, the relative growth rate of larvae after four days of feeding on transgenic maize leaves of the Muta4 transgenic maize line was compared to that after feeding on nontransgenic maize leaves. The larvae that fed on control, nontransgenic maize leaves exhibited a higher relative growth rate (0.125 mg/mg/day) than those that fed on transgenic maize (0.0769 mg/mg/day), as depicted in Fig 10A. *C*. *partellus* larvae fed transgenic maize leaves after four days exhibited up to 34% reduced mRNA expression of the chitinase gene compared to control larvae feeding nontransgenic maize leaves (Fig 10B). In the feeding assay, *C*. *partellus* larvae fed transgenic maize leaves exhibited significant mortality compared to the larvae fed control, nontransgenic maize leaves. Almost 90% of larvae that fed on nontransgenic leaves were found alive, whereas 42% of larvae that fed on transgenic leaves were found alive. The percentage mortality calculated by Abbott’s formula was 53% in larvae fed transgenic maize leaves. Our finding is similar to that of Rana et al. [21], who reported the suppression of chitinase in Lepidoptera as a target for lepidopteran control. We have further shown that transgenic maize expressing double-stranded chitinase suppresses *C*. *partellus* chitinase and can be employed for its control.

## Discussion

RNAi technology is currently employed as a viable means of controlling insect pests without disrupting other insects’ ecology because of its high target specificity. Thus, targeting the vital gene that causes detrimental effects on the insect [34] and effective dsRNA delivery are essential criteria for RNAi response efficacy. Spotted stem borer (*C*. *partellus*) is an invasive pest species that attacks maize and sorghum crops and causes significant yield losses [35, 36]. Previous studies suggested chitinase as an appropriate target for RNAi in insect pest management [37, 38]. In this study, we investigated the effectiveness of RNAi by targeting *C*. *partellus* chitinases. Chitinase is a family of 18 glycosyl hydrolases that breakdown glycosidic bonds in chitin. Chitin is a structural component of the insect trachea cuticle and the peritrophic matrix (PM) that lines the midgut lumen [10, 39]. However, several methods, such as oral feeding, microinjection, soaking, transfection, and host plant delivery, have evolved for dsRNA delivery. Efforts to develop host-induced gene silencing delivery methods have intensified because RNA-transgene plants continuously produce dsRNA that is eventually picked up by insects, and dsRNA transgene seeds can easily be made available to farmers. In this study, we used an oral route to deliver bacterially expressed *dsCHI* and purified *dsCHI* into the insect gut microenvironment to examine the knockdown efficiency of the targeted genes in *C*. *partellus*.

Several earlier studies have established that feeding-based RNAi can precisely induce an RNAi response in agricultural insects [40–44]. RNAi effectiveness generally depends on the sufficient concentration of ds/siRNA able to initiate the RNAi pathway. However, a higher concentration is most often used while implementing oral feeding assays. We discovered a significant knockdown of chitinase genes when we compared the transcript knockdown in larvae that were fed bacterially expressed dsRNA, purified dsRNA, and control. However, purified naked dsRNA exhibited low sensitivity. The reduction in transcript corresponded to the mortality recorded as larvae fed bacterially expressed dsRNA. The bacterially expressed dsRNA exhibited the lowest percent survival. However, we found that prolonged exposure of *C*. *partellus* larvae to naked *dsCHI* does not lead to enhanced silencing, which is ascribed to its quick degradation in the insect gut [45, 46]. Our results confirmed that direct exposure of naked dsRNA in *C*. *partellus* leads to rapid degradation of dsRNAs and affects dsRNA stability to induce RNAi. Recently, studies have shown that REase competes with Dicer-2 for targeted dsRNA, influences the unique total reads of target gene siRNAs, and, consequently, affects RNAi efficiency [47]. We have recently reported that protected dsRNA in *C*. *partellus* exhibited an optimal RNAi silencing effect [29]. Different physiological conditions in different tissues modulate enzyme activity, and different insects produce various dsRNA-degrading enzymes in different quantities [48]. In our study, the insects that were exposed to bacterially expressed dsRNA continuously exhibited significant knockdown as the time of exposure increased. Our result is similar to other reports that demonstrated that ingestion of bacterially expressed dsRNA led to reduction of *chitinase* transcript levels [18, 49, 50]. Protecting dsRNA from degradation by nucleases will be a better way of achieving optimum knockdown in *C*. *partellus*. Our results further strengthen the notion that rapid degradation of dsRNAs affects their ability to induce RNAi mechanisms and influence their stability. A comparison of dsRNA processing efficiency in Coleoptera, Lepidoptera, Diptera and Hemiptera indicated variability in dsRNA degradation efficiency by dsRNases [45, 46]. Efforts to protect dsRNA from nuclease degradation by silencing nucleases have been demonstrated to enhance dsRNA uptake in agricultural pests and to subsequently improve RNAi efficiency [51, 52]. Similarly, the knockdown of chitinase genes in *Chilo partellus* affects all metamorphic stages. This result indicated that chitinase is vital in the development of *C*. *partellus* and can be employed as a potential target for its control.

In this study, *dsCHI* was driven under the influence of the ubiquitin promoter. The transgenic maize lines exhibited different expression levels of *dsCHI*, which may be due to the difference in the site of integration or copy number. In our study, the results indicated that the larvae had a greater appetite for nontransgenic leaves than for transgenic leaves. This consequently inhibited the growth rate of the larvae that fed on the transgenic leaves. Furthermore, a significant reduction in chitinase transcript levels and an increase in mortality occurred in larvae fed transgenic leaves compared to those fed nontransgenic leaves. Our result agree with those of the previous report by Mamta et al., 2016 [19], where feeding on leaves of RNAi lines with reduced chitinase transcripts affected the overall growth and survival of *H*. *armigera*.

In conclusion, we have identified *C*. *partellus* chitinase as a potential target for RNAi-mediated control and demonstrated that oral delivery via bacteria and plants is a viable means of achieving *C*. *partellus* RNAi-mediated control. However, nanoparticles have been proposed as a novel delivery vehicle that protects dsRNA against RNases and circumvents the need for plant transformation. Nanoparticle delivery of *dsCHI* could be an efficient way of controlling *C*. *partellus* because it has been demonstrated to decrease dsRNA degradation and enhance uptake by insect cells, thus serving as a more reliable RNAi-based strategy [53–56].

## Supporting information

S1 File (DOCX)

S1 Raw images (PDF)
