# Supplementary material for: Host-induced silencing of the CpCHI gene resulted in developmental abnormalities and mortality in maize stem borer (Chilo partellus)
Source: PLoS One. 2023 Feb 6;18(2):e0280963. doi: 10.1371/journal.pone.0280963 (PMC9901779; doi:10.1371/journal.pone.0280963)
Supplement: S1 File — (DOCX) [file pone.0280963.s001.docx]

# **Host-induced silencing of *CpCHI* gene occasioned developmental abnormalities and mortality in Maize stem borer (***Chilo partellus*)

Authors: Olawale Samuel ADEYINKA^1,2^*, Idrees Ahmad Nasir^1^ and Bushra Tabassum^1,3^

Affiliation: ^1^Centre of Excellence in Molecular Biology, University of the Punjab, Lahore-Pakistan 53700.

^2^Department of Chemistry, Physics and Atmospheric Sciences Jackson state University, Jackson, MS 39217, USA.

^3^School of Biological Sciences, University of the Punjab, Lahore, Pakistan

*Correspondence: E-mail: [adeyinka.olawale@gmail.com](mailto:adeyinka.olawale@gmail.com) ORCID: 0000-0003-1430-9538


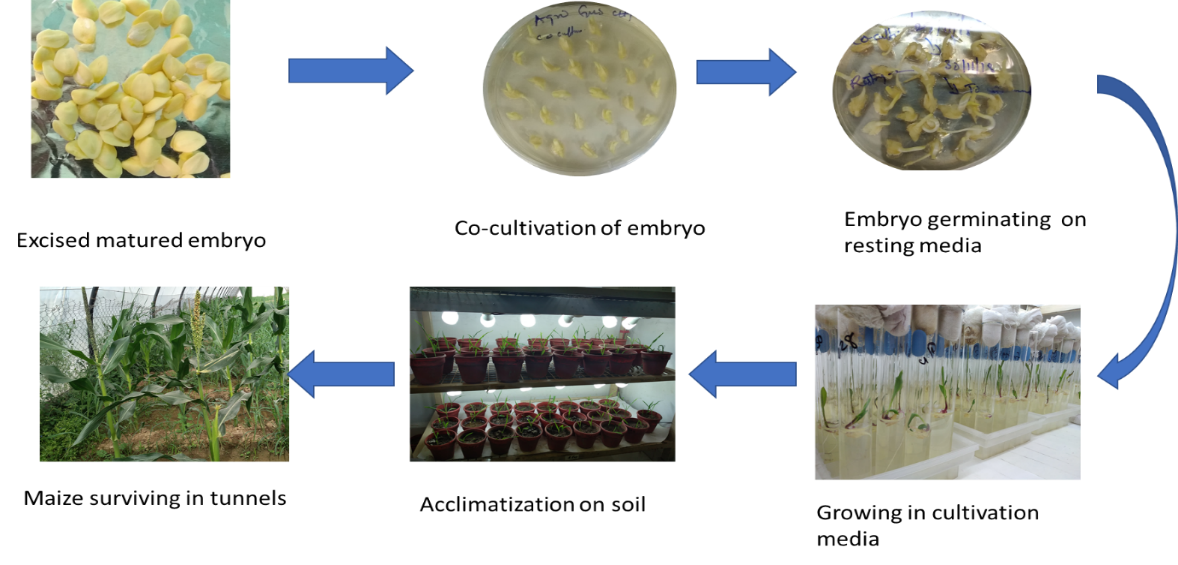


S1 Fig. The schematic transformation steps of mature maize embryo with positive Agrobacterium harbouring the Recombinant pCAMBIA plasmids


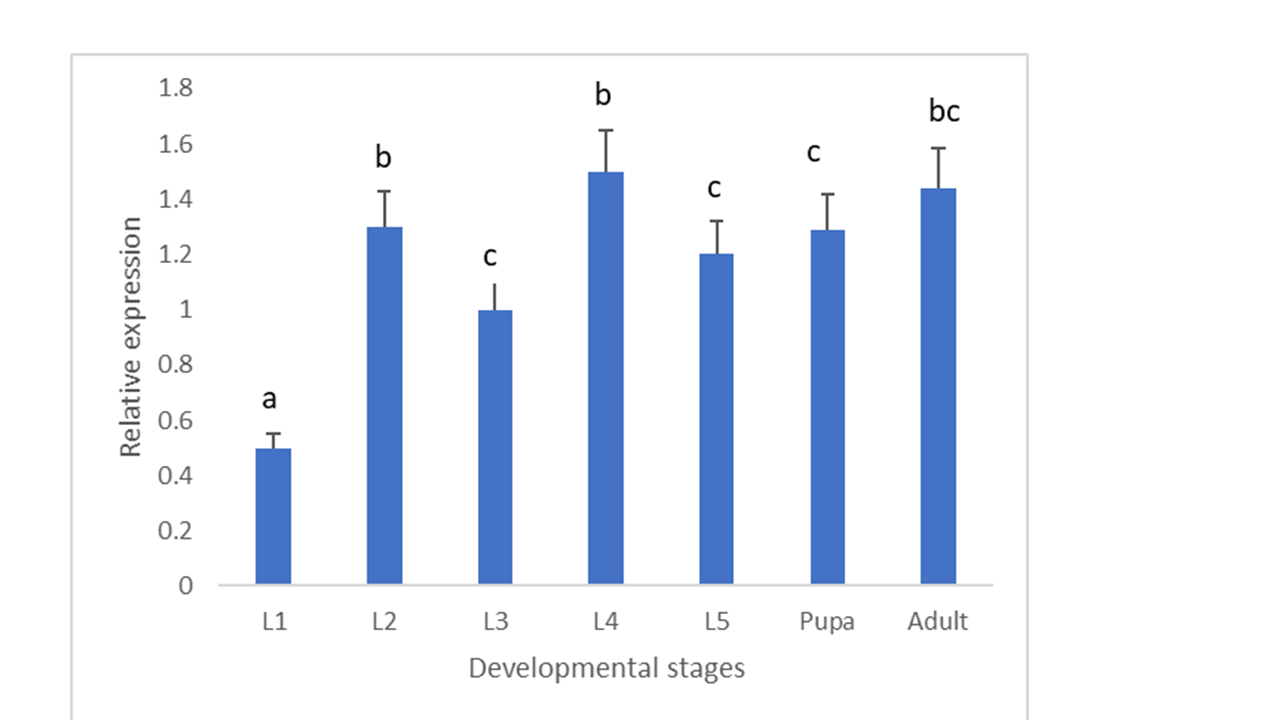


a

**S2 Fig. Temporal expression profiles of the *C. partellus* chitinase gene across all developmental stages**. Each data point represents the mean ± standard error of results from three technical replicates.

| 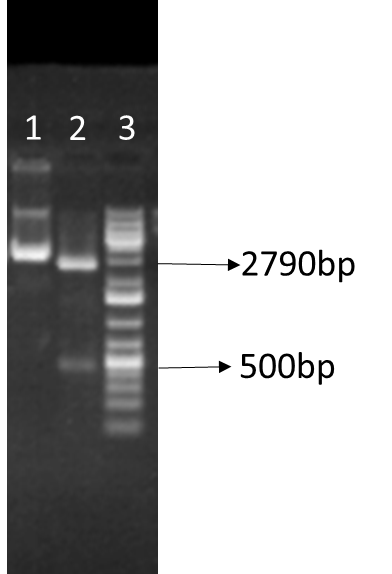  **S3A Fig.** Restriction digestion of positive clones confirming the chitinase gene insertion in L4440 vector. Lane 1; undigested L4440-*dsCHI*, Lane 2; digested L4440-*dsCHI*, Lane 3; 1kb plus ladder. | 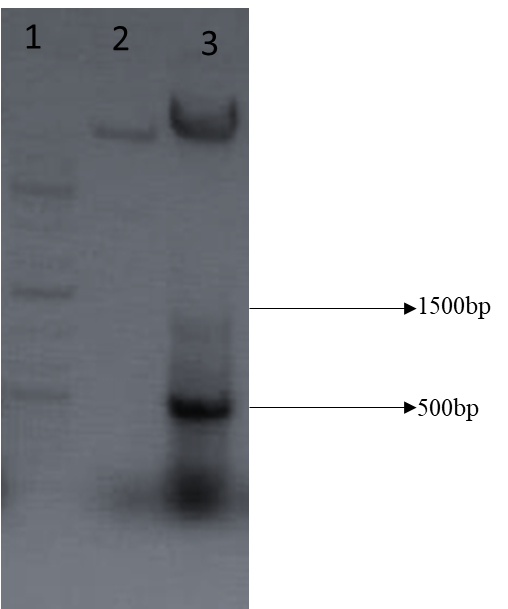  S3B Fig. Synthesis of double-stranded chitinase from IPTG induced HT115 bacterial culture. Lane 1; 1kb ladder, Lane 2; uninduced HT115-*dsCHI* Lane 3; induced HT115-*dsCHI*. |
| --- | --- |

A
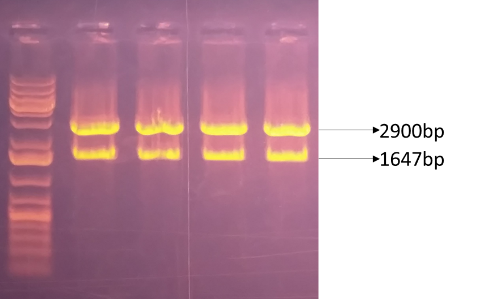
B
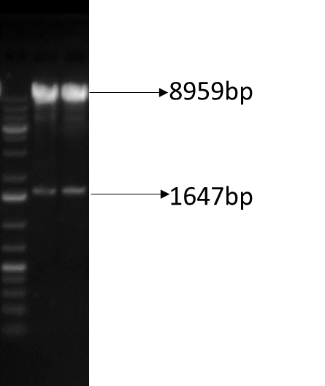
C
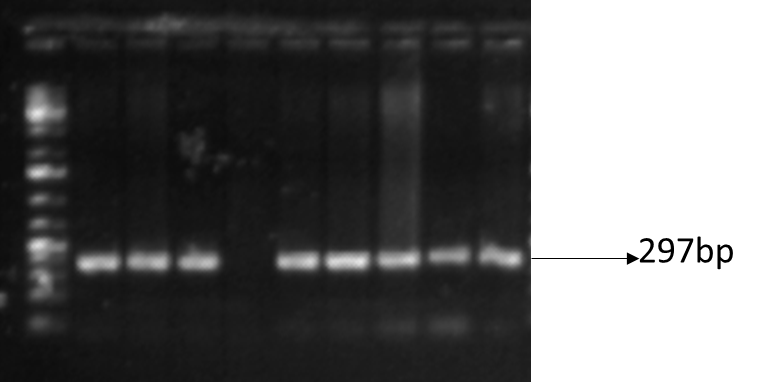


S4A Fig. Restriction confirmation of *dsCHI* in pUC19 backbone vector. S4B Fig. Confirmation of *dsCHI* in pCAMBIA vector. S4C Fig. Confirmation of transformed *dsCHI* via colony PCR


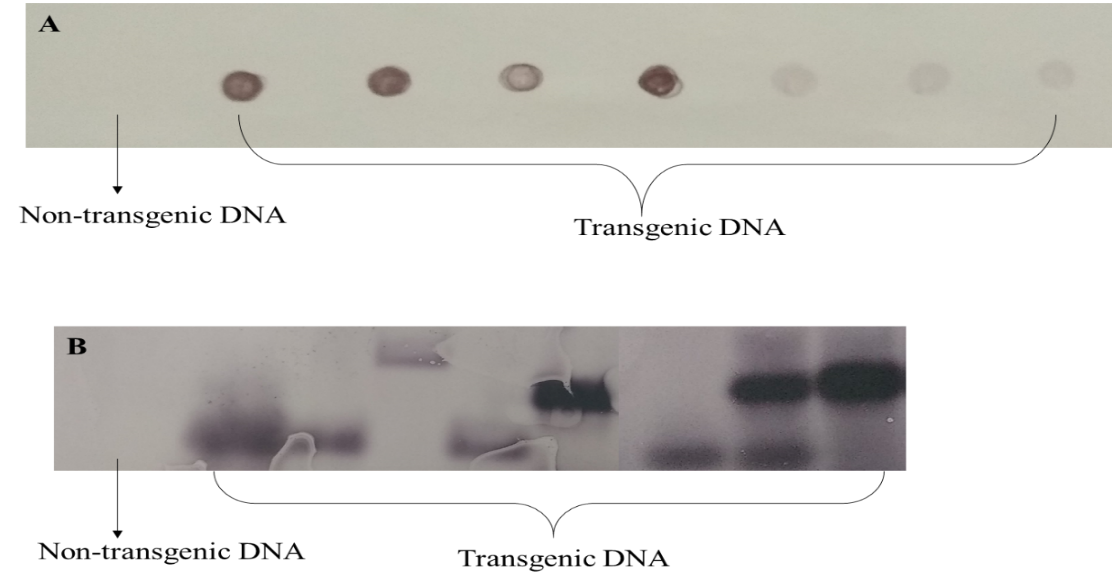


**S5A Fig. Dot blot analysis and Southern analysis** **blot analysis for denatured DNA from non-transgenic and transformed maize. S5B Fig. Southern blot analysis of transformed plants indicated hybridization signal in transformed maize while no signal was dictated in the control plant**
