# Supplementary figures and images for: Host-induced silencing of the CpCHI gene resulted in developmental abnormalities and mortality in maize stem borer (Chilo partellus)
Source: PLoS One. 2023 Feb 6;18(2):e0280963. doi: 10.1371/journal.pone.0280963 (PMC9901779; doi:10.1371/journal.pone.0280963)

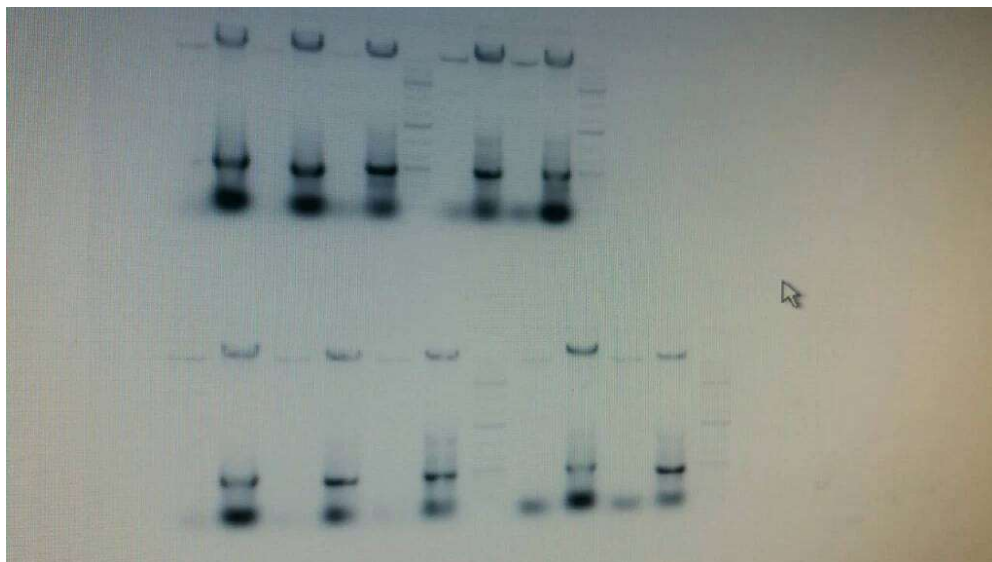

S3\_raw\_images

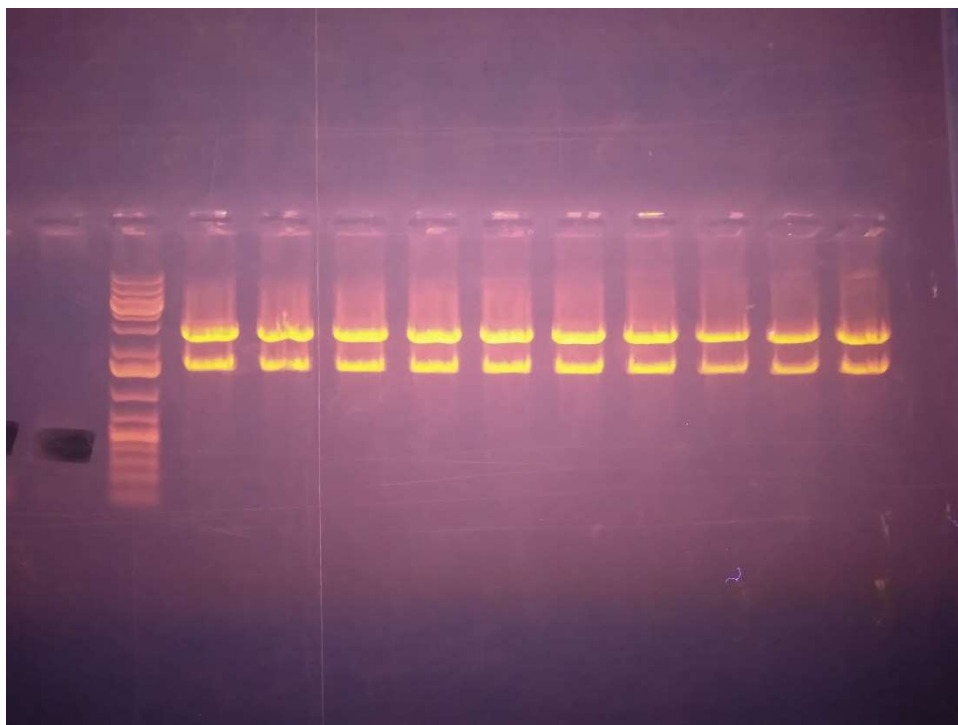

S4 (A)\_raw\_images

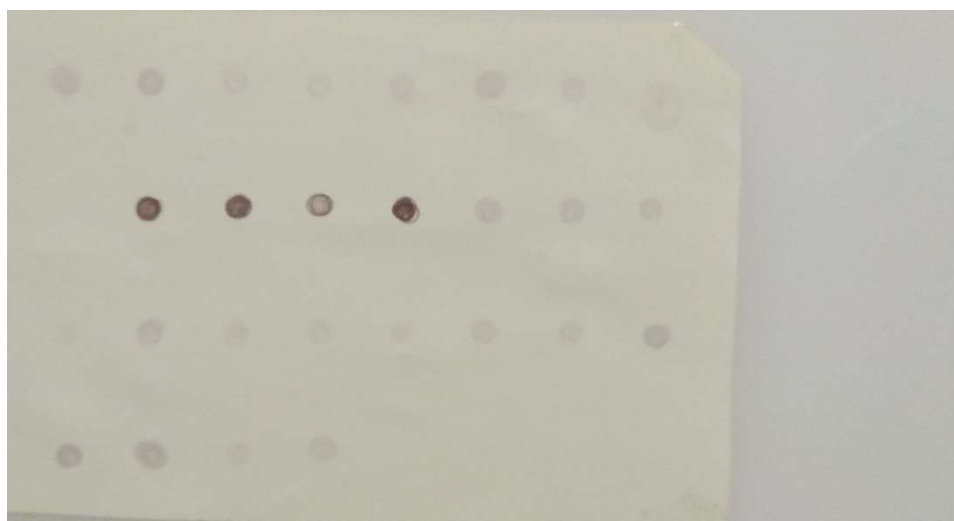

S5 Fig. (A) \_raw\_images

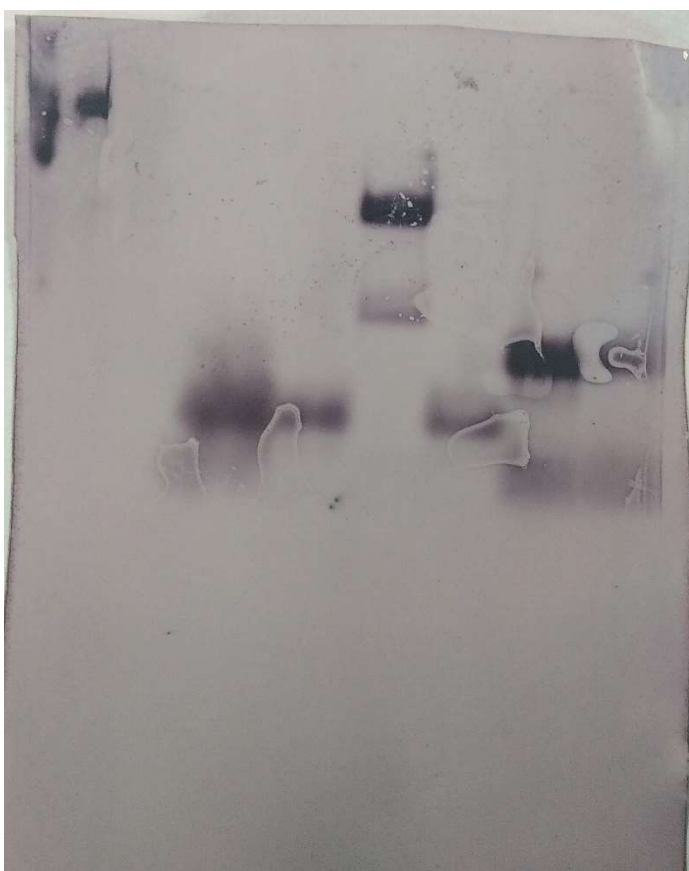

S5 Fig. (B) \_raw\_images

Supplement: S1 Raw images — (PDF) [file pone.0280963.s002.pdf]
